# Use of the General Movements Assessment for the Early Detection of Cerebral Palsy in Infants with Congenital Anomalies Requiring Surgery

**DOI:** 10.3390/jcm8091286

**Published:** 2019-08-22

**Authors:** Cathryn Crowle, Alison Loughran Fowlds, Iona Novak, Nadia Badawi

**Affiliations:** 1 The Children’s Hospital Westmead, Sydney 2145, Australia; 2 Faculty of Medicine, The University of Sydney, Sydney 2050, Australia; 3 Cerebral Palsy Alliance, Sydney 2100, Australia

**Keywords:** general movements, congenital anomalies, cerebral palsy, early detection

## Abstract

The general movements (GMs) assessment is recognised as one of the most important tools in the early detection of cerebral palsy (CP). However, there remains a paucity of data on its application to infants with congenital anomalies requiring surgery. This was a prospective study of 202 infants (mean gestation 38 weeks, SD 2.2) who had undergone major surgery for congenital anomalies in the neonatal period. Infants were assessed at three months of age (mean 12 weeks, SD 1.6) and GMs videos were independently rated by three clinicians, two blinded to clinical details. Developmental follow-up was at three years of age. Of the twenty-five infants (9%) rated as having an absence of fidgety movements, 22 were seen at 3 years, and 17 had an abnormal outcome: 11 with CP, and 6 with a developmental disability. Infants with absent fidgety movements were 21.5 (95% CI 7.3–63.8) times more likely to have an abnormal outcome including CP. None of the infants with normal fidgety movements had a diagnosis of CP and 86% were assessed to be developing normally. The GMs assessment has predictive value for cerebral palsy and neurodevelopment for infants with congenital anomalies, and should be incorporated into routine follow-up to facilitate early referral.

## 1. Introduction

The general movements (GMs) assessment is recognised to be one of the best tools for the early prediction of neurodevelopmental outcomes in infancy, especially cerebral palsy [1,2]. This assessment involves a visual/Gestalt perspective of an infant’s spontaneous movements which are recorded for several minutes when the infant is in a calm, awake state [3]. Training is required to rate an infant’s movements as normal or abnormal, and then further into descriptive categories. This assessment can be used in the preterm and term age to assess an infant’s writhing movements, and then again between 7–20 weeks to determine the presence of ‘fidgety’ movements. Fidgety movements are small movements of moderate speed and variable acceleration seen everywhere in the body, and are present continuously or intermittently in typically developing infants between 9 to 15 weeks post-term age [4]. Absent fidgety movements have a strong association with the prediction of cerebral palsy (97–98% sensitivity, 89–91% specificity) [2,5].

The recent paper by Novak and colleagues [6] recommends the GMs assessment for the early detection of infants at risk of cerebral palsy based on the available evidence. Although the recommendations include use of the GMs assessment with infants born with congenital anomalies, less has been published in this area, with a large proportion of GMs research focusing on the preterm infant population and those with encephalopathy. We know the GMs assessment is feasible to use in the NICU following neonatal surgery [7] and that a small but significant group of infants demonstrate absent fidgety movements [8]. Previous research by our group showed a strong prediction for CP at 12 months of age with sensitivity and specificity reported as 100% and 96% respectively [9]. However, a rating of normal fidgety movements at three months of age did not guarantee a normal developmental outcome at 12 months of age in this cohort, as almost half the infants showed delayed development at this early stage of life. This was not unexpected, given the population often required multiple or lengthy admissions, and may have undergone subsequent surgeries.

The aim of this paper was to determine the effectiveness of the GMs assessment in predicting neurodevelopmental outcomes, including cerebral palsy, at three years of age in a cohort of infants with congenital anomalies requiring major surgery in the neonatal period. Secondly, the paper aimed to determine whether the presence of normal fidgety movements was a better predictor of normal motor outcome at 3 years than at 12 months of age. This is important for families who are often anxious following their infant’s stay in the neonatal intensive care unit, particularly following major surgery [10,11].

## 2. Materials and Methods

This was a prospective study, investigating the use of the GMs assessment, to predict neurodevelopment in infants with congenital anomalies who required major surgery in the neonatal period for either cardiac or non-cardiac related conditions. Infants were recruited from a tertiary level NICU at the Children’s Hospital at Westmead between 2013 and 2015, where the majority of neonatal surgery in the state of New South Wales is performed. For the cardiac surgery group, major surgery included infants undergoing both open and closed cardiac surgery, but excluded infants who only had patent ductus arteriosus ligation. For the non-cardiac surgery group, major surgery was defined as surgery requiring the opening of a body cavity, such as a laparotomy or thoracotomy. Ethics approval for the study was obtained through The Sydney Children’s Hospital Network, Human Research Ethics Committee (LNR/12/SCHN/513) and informed consent was obtained from all parents/carers.

### 2.1. Participants

This prospective study initially enrolled a group of 304 infants born at term age (mean gestation 38 weeks, SD 2.1) with congenital anomalies requiring surgery. Infants were eligible for the study if they had undergone major surgery in the first 30 days of life and were enrolled for developmental follow-up through the clinic. The development clinic follows all infants undergoing major surgery through the unit. This includes cardiac anomalies such as coarctation of the aorta, pulmonary stenosis, truncus arterious, or hypoplastic left heart syndrome, and non-cardiac anomalies such as abdominal wall defects, trachea-esophageal fistula, or diaphragmatic hernia. Infant characteristics and a full list of congenital anomalies requiring surgery are detailed in Table 1.

### 2.2. Procedure

All infants had a GMs assessment at their first multidisciplinary clinic appointment, scheduled for 12 weeks post-term age, along with the Bayley Scales of Infant and Toddler Development III (Bayley-III) and clinical/neurological examination. GMs videos were recorded by a GMs trained occupational therapist according to the procedures outlines by the General Movements Trust, with infants positioned in supine, in a quietly awake state [12]. Videos were independently scored according to Prechtl’s method by three advanced trained clinicians, two blinded to clinical details and external to the hospital site. Infants were rated as demonstrating normal, abnormal or absent fidgety movements.

Follow-up appointments were scheduled for three years of age within the development clinic. At this appointment, a physiotherapist and occupational therapist trained and experienced in developmental assessment, administered and scored the Bayley-III without ready access to the GMs results. Medical and neurological review was conducted by the neonatologist or senior neonatal trainee. Throughout the appointment, the team observed the movement quality and any clinical signs of cerebral palsy such as abnormal movement and posture (spasticity, dyskinesia or ataxia), or asymmetry of hand and arm function. A small number of infants (*n* = 7) did not attend the development clinic appointment at 3 years of age, as they had a confirmed diagnosis of CP following diagnosis by a paediatric neurologist or neonatologist, and were being seen in another service. This information was added to the database in order to monitor the outcomes of all infants in the study group.

### 2.3. Statistical Analysis

To describe the utility of the GMs assessment to predict neurodevelopment in infants with congenital anomalies requiring surgery, an odds ratio for an abnormal outcome following a rating of absent fidgety movements was calculated. The sensitivity and specificity for absent fidgety (as test positive) for the prediction of CP, and the sensitivity and specificity for normal fidgety (as test positive) for normal development were also calculated. Statistical analysis was completed using SPSS and Stata.

Infants were considered to be demonstrating normal development if they scored within the average range across all areas on the Bayley-III according to test norms (mean of 10 and SD of 3). Mild delay was defined as scores between 1 and 2 standard deviations below the mean in one or two domains (cognition, language or motor). However, infants who showed below average scores only in the domain of language were not coded as having developmental delay, as many children in this cohort were exposed to languages other than English at home, and our clinical experience has found these children often go on to have a normal outcome. Global delay or developmental disability was used to describe infants who scored below the average range across all areas (motor, cognition, language) or who had received a diagnosis such as autism spectrum disorder.

It was not appropriate to statistically compare the Bayley-III scores between the group of infants who had normal fidgety GMs to the group who had absent fidgety GMs, as 7 out of the 11 infants with CP did not have a Bayley-III administered as they had been discharged from clinic.

## 3. Results

**Figure 1:**
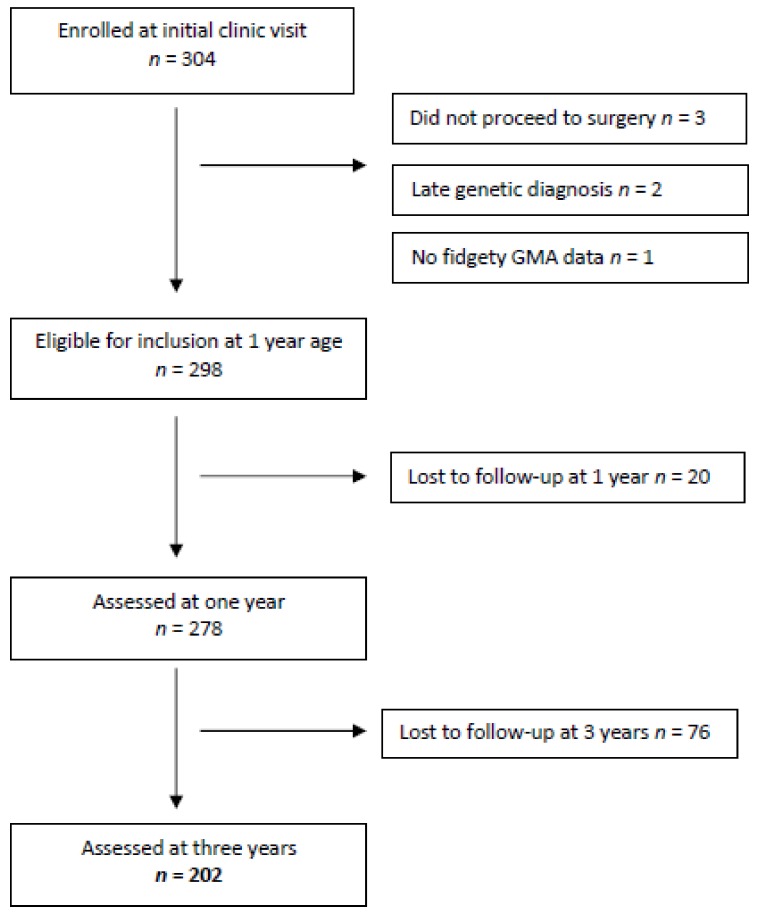
Study sample.

There were 304 infants enrolled in the study at their first clinic appointment held at a mean age of 12 weeks (SD 1.6). From this group, 278 infants were assessed at one year of age and considered eligible for follow-up at age three (see Figure 1). Of this group, 149 infants (54%) had undergone cardiac surgery, 123 non-cardiac surgery (44%), and 6 infants had both types of surgery (2%).

**Figure 2:**
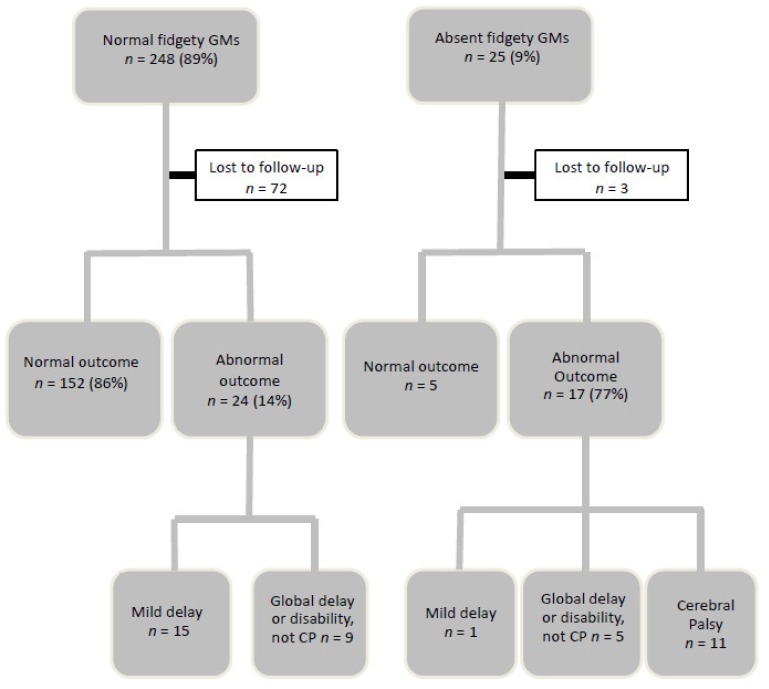
Outcomes at 3 years of age following normal and absent fidgety movements.

Follow-up was at three years of age (mean 38.1 months, SD 1.8). There were no families who refused consent or opted to withdraw from the study, however, there were 76 infants (27%) lost to follow-up at age three, leaving a group of 202 infants. The GMs results at three months post-term age showed that the majority of infants had normal fidgety movements (*n* = 248, 89%); 25 (9%) had absent fidgety movements, and 5 (2%) had abnormal fidgety movements. These results and the outcomes at three years of age are illustrated in Figure 2.

### 3.1. Normal Fidgety GMs at 3 months.

There were 248 (89%) infants with normal fidgety movements and 176 were seen at three years of age. There were *n* = 72 lost to follow-up due to geographic location (declined appointment due to distance), relocation interstate or overseas, or an inability to make contact with the family. None had a diagnosis of CP and the majority *n* = 152 (86%) were assessed to be developing normally on the Bayley Scales of Infant and Toddler Development, third edition. The mean group subtest scores on the Bayley-III were all within the average range: Cognition 9.47; receptive communication 10.17; expressive communication 9.61; fine motor 10.06; gross motor 8.86. This is in contrast to the much lower proportion assessed to be developing normally at 12 months of age following a rating of normal fidgety general movements, which was 52% (*n* = 130).

There was no significant difference in the length of stay in the neonatal intensive care unit when comparing developmental outcome at one or three years of age in this group of infants with normal fidgety movements. However, the need for a subsequent surgery was associated with the outcome at one year of age X^2^ = 9.206 (*p* = 0.003); unadjusted odds ratio = 2.4.

There were 24 infants (14%) who had an abnormal outcome at three years of age: 8 infants with gross motor delay only, with a mean gross motor scaled score of 4.5; and 7 infants with mild developmental delay (language and motor) as assessed on the Bayley III. There were 9 infants with global delay, scoring below the average range across all areas (mean group scaled scores: Cognition 4.5, receptive language 2.6; expressive language 3.2; fine motor 5; and gross motor 4.3).

A normal fidgety GMs result at three months of age had a sensitivity and specificity of 97% and 41% respectively for the prediction of normal development.

### 3.2. Absent Fidgety GMs at 3 months

There were 25 infants (9%) with absent fidgety movements and 22 were seen at three years of age (*n* = 2 lost to follow-up, *n* = 1 deceased). Of these, 17 (77%) had an abnormal outcome: 11 with CP (6 with spastic hemiplegia, 2 with spastic diplegia, and 3 with spastic quadriplegia); 6 with developmental disability, including CHARGE syndrome, vision and hearing impairment, and hypotonia with undiagnosed genetic disorders. Infants with absent fidgety movements were 21.5 (95% CI 7.3–63.8) times more likely to have an abnormal outcome including CP. Despite low numbers diagnosed with CP in this population (6%), sensitivity and specificity for CP were high (sensitivity = 100%, specificity = 94%).

The group of infants with an abnormal outcome had high levels of rater agreement on their GMs assessment; 59% (*n* = 10 from 17 infants) had been rated as having absent fidgety movements by all three raters. Furthermore, all infants with spastic quadriplegia classified as GMFCS IV–V, had received a unanimous GMs rating. In contrast, for those infants who were developing normally at age three (*n* = 5), none had been rated as having an absence of fidgety movements by all three raters.

### 3.3. Abnormal Fidgety GMs at 3 months, n = 5

There were five infants rated as having abnormal fidgety movements. These are fidgety movements that are of a larger amplitude and are rarely seen [12]. Only one infant in this group was lost to follow-up. At three years of age, three infants were developing normally and one had a diagnosis of Autism Spectrum Disorder.

## 4. Discussion

To our knowledge, this is the first paper to systematically examine the three year outcomes of a large group of infants with congenital anomalies requiring surgery following use of the GMs assessment. The GMs assessment is a highly sensitive and specific tool that is able to accurately identify premature infants at high risk of cerebral palsy at three months of age, facilitating early referral for intervention [1,2,5]. Our study shows that the GMs assessment is also highly sensitive and specific for the term born infant surgical population for the prediction of neurodevelopment, including cerebral palsy, at three years of age.

For infants with normal fidgety movements, the test was not as sensitive or specific, with a proportion continuing to have developmental delay at three years of age. However, in this group there was a reduction in the number that demonstrated developmental delay at three years of age, compared to the number showing delay at age one, suggesting a number of infants had ‘caught up’ to their peers. The proportion showing delays in development at age one was 48%, however, by three years of age this had reduced to 14%, and there were no infants with cerebral palsy. A rating of normal fidgety movements is, therefore, reassuring for parents of infants who have undergone major surgery for congenital anomalies, and who are often anxious regarding their infant’s development. 

In this group of infants with normal fidgety movements, the length of stay in the neonatal intensive care unit was not associated with developmental outcome. However, the need for subsequent surgery requiring an additional hospitalization or transfer to another ward, was shown to increase the likelihood of a poor outcome at one year of age. Further research is needed to investigate the impact of the overall hospital length of stay, parental stress, maternal education and level of support, as these have been associated with developmental outcomes in infants following major surgery [13].

The GMs assessment was useful in clinical decision making in the development clinic, as it facilitated the early identification of the high risk of cerebral palsy at three months of age and ensured these infants were referred into more specialised services. Early intervention in New South Wales can primarily be accessed in two ways. The first is community based Allied Health services (physiotherapy, occupational therapy and speech pathology) operated by the Department of Health, usually for short blocks of therapy to address delays in a specific area of development. The second is via the National Disability Insurance Scheme (NDIS) which is aimed at supporting people with a lifelong disability. Families with an NDIS funding package are able to access specialised service providers such as the Cerebral Palsy Alliance. This is important, as we know that cerebral palsy specific early intervention is the recommended approach for infants identified as being at risk of CP [6].

Although not all infants with absent fidgety movements went on to have CP, a large proportion had a poor outcome (77% had an abnormal outcome including CP). It is important to note that although the GMs assessment did not fail to identify any infants with CP, caution should be applied in interpreting absent fidgety movements at three months of age as a guarantee to develop CP. There may be other developmental disabilities, which is not surprising as neonates requiring major surgery can have underlying genetic conditions. It has been reported that genetic disorders or syndromes may be found in up to 30% of pediatric patients with congenital heart disease [14].

The GMs assessment was universally accepted among parents and was feasible to carry out within the multidisciplinary development clinic. It would be interesting to investigate whether the age at administration would change the accuracy of the detection of poor neurodevelopment, as these videos were all taken at 12 weeks post-term age. An assessment conducted later in the period of fidgety movements, or a subsequent GMs assessment may have improved the results.

Prediction of outcomes was strengthened when strong inter-observer agreement was reached, particularly for a rating of ‘absent fidgety’ GMs. Previous investigation by our group into the strength of inter-observer agreement when scoring GMs in this population, showed excellent agreement and reliability using experienced, advanced trained raters [15]. Therefore, team review of GMs videos is recommended for this population to enhance the reliability of scoring.

This study was limited by the loss to follow-up of infants from age one to three. Infants who are offered follow-up in our clinic are drawn from a large geographic area of the state of New South Wales which makes return for follow-up difficult in some cases. Appointments are also limited by sufficient staffing and funding for the outpatient follow-up clinic. Financial and resourcing aspects are often a barrier to ideal follow-up, especially of cardiac and surgical infants internationally, as resources tend to be diverted to the more well established follow-up programs of extremely preterm infants, despite the knowledge that infants with congenital anomalies requiring surgery are considered to be a high risk group [6,16].

Another consideration is the use of the optimality scoring concept for the GMs assessment [17] to potentially enhance the prediction of outcomes and, therefore, assist in clinical decision making. There were a large number of infants in this study with normal fidgety movements that were also noted to be sub-optimal in their movement quality. Although observations of concurrent age-related motor repertoire were taken into account in recommendations for further management, optimality scoring was not in regular use in the development clinic at the time of this study. The use of the optimality score should thus be investigated further, specifically in a cohort of infants with normal fidgety movements, as lower optimality scores or an abnormal concurrent motor repertoire have been associated with an increased risk of minor neurological dysfunction [18,19].

Ideally, the GMs assessment should be used in conjunction with other evidence-based tools such as the Hammersmith Infant Neurological Examination (HINE) and neuroimaging, as recommended in the international guidelines on the early detection of cerebral palsy [6]. Use of these tools together may have further improved the prediction of neurodevelopmental outcome, and the HINE has subsequently been added as a routine assessment in our Development Clinic for all infants at 3–4 months of age.

Future research investigating outcomes for cardiac and surgical infants at age five and eight is recommended because different types of developmental disability become apparent during certain age periods [14]. The complexity of self-care and school related tasks increases with age and some infants who may not have shown signs of early delay may go on to have difficulties that affect their function and participation at home and school. Further analysis of the severity of developmental delay, and its relation to therapy interventions in infants with normal and/or absent general movements, would also be helpful in counseling parents in both the initial admission and in subsequent follow-up clinics.

## 5. Conclusions

The GMs assessment is predictive for cerebral palsy at three years of age in infants with congenital anomalies requiring cardiac and non-cardiac surgery. It has similarly strong predictive value in this unique population as compared to other high risk infant groups such as those born preterm. Reassuringly for parents, 86% of infants with normal fidgety movements demonstrated normal development by the age of three years. The GMs assessment is an essential tool that should be incorporated into routine standardised developmental follow-up for infants with congenital anomalies to facilitate early referral to specialised community therapy services, and provide increased accuracy in counselling about long term developmental outcomes. Team scoring is recommended as this is a difficult population to score and accuracy is improved with higher inter-observer agreement. Further research is needed to clarify the relationship of the GMs assessment to longer term developmental outcomes in this highly vulnerable population.

## Figures and Tables

**Table 1:** Infant characteristics.

*n* = 278			
Male		165 (59%)	
Gestational age (weeks)		Mean 38.1 (SD 2.1)	
		Median 39 (range 30–41)	
Birth weight (grams)		Mean 3104.7 (SD 651.9)	
		Median 3100 (range 1240–4970)	
Age at GMA (Post-term age in weeks)		Mean 12.4 (SD 1.6)	
Age at 1 year follow-up (corrected age in days)		Mean 372 (SD 13)	
Age at 3 year follow-up (months) (*n* = 202)		Mean 38.1 (SD 1.8)	
Cardiac surgery	149 (54%)	Non-cardiac surgery	123 (44%)
Left Heart obstructive lesion	52		
Hypoplastic left heart Syndrome	9	Abdominal wall defects/cloacal anomalies	20
Coarctation of the aorta	16	Gastroschisis	16
Coarctation with VSD	11	Omphalocele	2
Aortic stenosis	2	Exomphalos	2
Dysplastic aortic arch/valve	6		
Heart block/pacemaker	2		
Atrio Ventricular Septal Defect	6		
TGA/truncus arteriosus	38	Intestinal Atresia	11
Patent ductus arteriosus	2	Meconium ileus	3
Right Heart Obstructive Lesion	38		
Pulmonary atresia/stenosis	24	Malrotation	7
TAPVR	3	Imperforate anus/anorectal malformation	14
Tetralogy of Fallot	4	Tracheo-esophageal fistula/oesophageal atresia	14
Tricuspid atresia	3	Hirschsprung’s disease	16
Double outlet right ventricle	4		
		Head/neck pathology	11
		Pierre Robin sequence	3
		Anterior glottic web	1
Complex cardiac	15	Occipital encephalocele	1
Other	4	Bilateral choanal atresia	1
		Depressed parietal bone	1
		HIE, PVL, hydrocephalus	3
		Vein of Galen	1
		Renal/urological	5
		Pyloric stenosis	2
		VACTERL	3
		Other	4
Both types surgery	6 (2%)		
